# Suitable Days for Plant Growth Disappear under Projected Climate Change: Potential Human and Biotic Vulnerability

**DOI:** 10.1371/journal.pbio.1002167

**Published:** 2015-06-10

**Authors:** Camilo Mora, Iain R. Caldwell, Jamie M. Caldwell, Micah R. Fisher, Brandon M. Genco, Steven W. Running

**Affiliations:** 1 Department of Geography, University of Hawai'i at Manoa, Honolulu, Hawai'i, United States of America; 2 Hawai'i Institute of Marine Biology, University of Hawai'i at Manoa, Honolulu, Hawai'i, United States of America; 3 School of Forestry, University of Montana, Missoula, Montana, United States of America; University College London, UNITED KINGDOM

## Abstract

Ongoing climate change can alter conditions for plant growth, in turn affecting ecological and social systems. While there have been considerable advances in understanding the physical aspects of climate change, comprehensive analyses integrating climate, biological, and social sciences are less common. Here we use climate projections under alternative mitigation scenarios to show how changes in environmental variables that limit plant growth could impact ecosystems and people. We show that although the global mean number of days above freezing will increase by up to 7% by 2100 under “business as usual” (representative concentration pathway [RCP] 8.5), suitable growing days will actually decrease globally by up to 11% when other climatic variables that limit plant growth are considered (i.e., temperature, water availability, and solar radiation). Areas in Russia, China, and Canada are projected to gain suitable plant growing days, but the rest of the world will experience losses. Notably, tropical areas could lose up to 200 suitable plant growing days per year. These changes will impact most of the world’s terrestrial ecosystems, potentially triggering climate feedbacks. Human populations will also be affected, with up to ~2,100 million of the poorest people in the world (~30% of the world’s population) highly vulnerable to changes in the supply of plant-related goods and services. These impacts will be spatially variable, indicating regions where adaptations will be necessary. Changes in suitable plant growing days are projected to be less severe under strong and moderate mitigation scenarios (i.e., RCP 2.6 and RCP 4.5), underscoring the importance of reducing emissions to avoid such disproportionate impacts on ecosystems and people.

## Abstract 2

While ongoing climate change can increase the number of days above freezing, changes in other climatic conditions will lead to fewer days when plants can grow, which in turn will affect biodiversity and people. See the Synopsis.

## Author Summary

Ongoing greenhouse gas emissions can alter climate suitability for plant growth, in turn affecting biological and social systems. Using the latest generation of available climate projections we show that there will be fewer days with suitable climates for plant growth, despite an increase in days above freezing. This decline in suitable plant growing days is due to interactions among unsuitable temperatures, light, and water availability. Our analysis shows that reductions in suitable plant growing days will be most pronounced in tropical areas and in countries that are among the poorest and most highly dependent on plant-related goods and services. Changes in suitable plant growing days will be less severe under strong and moderate mitigation scenarios, highlighting the importance of reducing emissions to ameliorate the biological and social impacts of these changes.

## Introduction

Plant growth is a fundamental biological process that is strongly controlled by climate variables [1–6]. Plant productivity influences the functioning of ecosystems [7], fuels the global food web [8], and is the foundation for some of the most diverse habitats in the world [9]. Vegetation also sustains humanity [10–12], directly providing oxygen, food, fiber, and fuel (e.g., an estimated ~30%–40% of the biosphere production is currently consumed or coopted by humans [13–18]) and indirectly supporting livelihoods through jobs and revenue [19]. However, plant growth is strongly limited by climate variables such as air temperature, water availability, and solar radiation [1–4,20–22], which are changing in response to ongoing climate change [23–26]. These changes are concurrent with a greater human demand on the planet’s resources, which could further stress natural ecosystems and lead to shortages in important goods and services [13,15,27–29]. While there have been considerable advances in understanding the extent to which individual [30] and multiple [4,6,31] climate variables limit plant growth [e.g. 20,23–25,32–34], comprehensive analyses integrating climate, biological, and social sciences are less common. Here we provide a global-scale perspective, using climate model projections (S1 Table) and available socioeconomic and ecological data (S2 Table), to assess how projected climate change will affect the suitability of the planet for plant growth and evaluate potential implications of these changes for ecosystems and people.

## Results and Discussion

**Fig 1:**
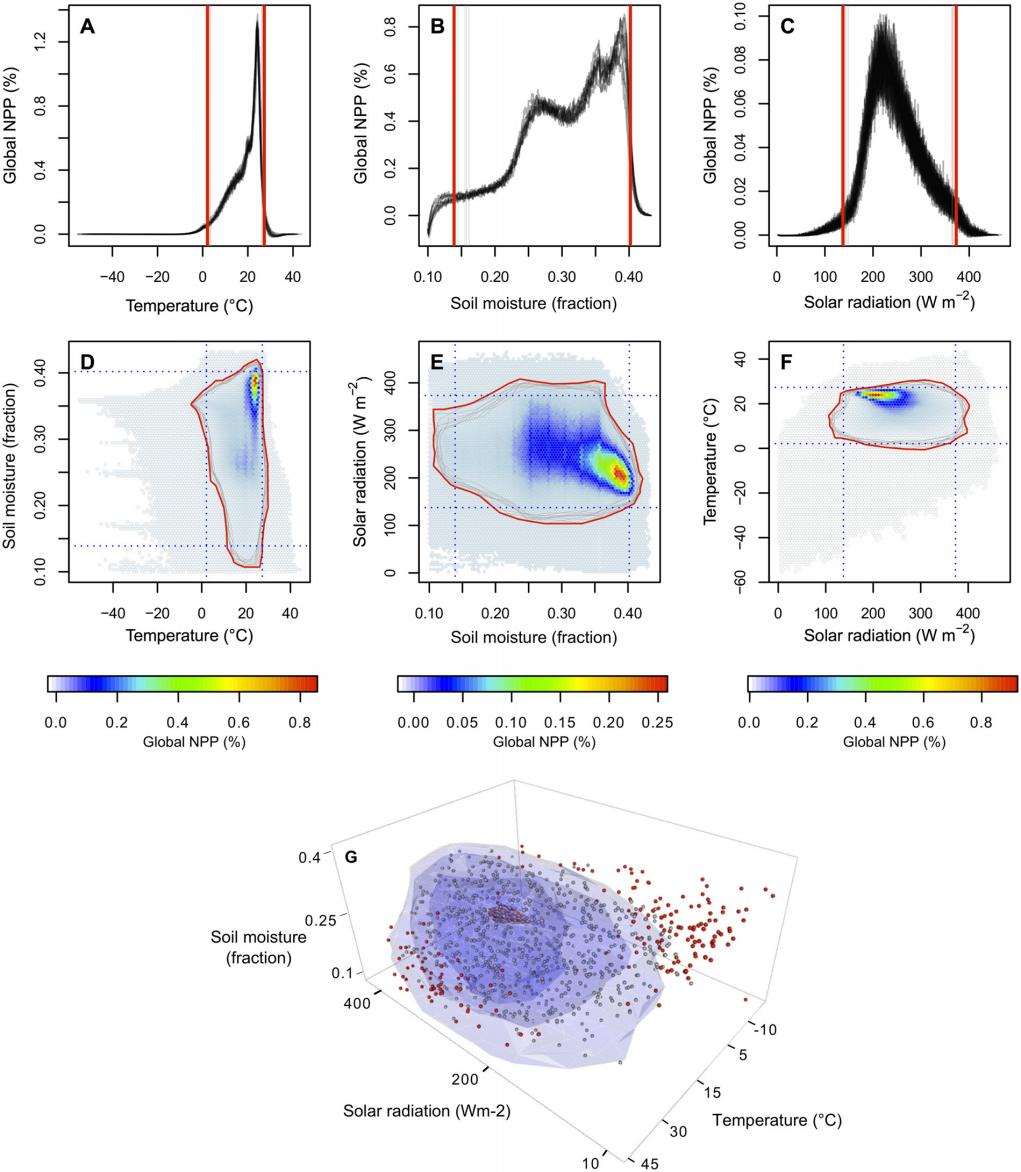
Climatic ranges for plant growth. Global vegetative matter produced (i.e., MODIS NPP, http://neo.sci.gsfc.nasa.gov/view.php?datasetId=MOD17A2_E_PSN) along gradients of temperature (A), soil moisture (B), solar radiation (C), and the interactions of these three variables (D–G). Climate data were obtained from National Centers for Environmental Prediction (NCEP) Reanalysis Daily Averages (http://www.esrl.noaa.gov/psd/cgi-bin/db_search/DBSearch.pl?Dataset=NCEP+Reanalysis+Daily+Averages+Surface+Flux&group=0&submit=Search). Grey lines in plots A–F indicate the climatic conditions that surround 95% of the global NPP each year between 2004 and 2013. Red lines encompass all of the yearly boundaries and define the climatic thresholds used in our analysis. A suitable plant growing day was defined as any day falling within these climatic thresholds. Points in plot G are a random subset (i.e., 1,000 points) of global climate conditions and resulting NPP (grey points indicate positive NPP/growth, and red points indicate negative NPP/respiration). As illustrated, climatic conditions occurring beyond the estimated global thresholds have commonly resulted in plant respiration. See also S1 Fig. Data are provided in S1 Data.

To assess the future limiting roles of temperature, water availability, and solar radiation on plant growth, we calculated changes in the number of days in a given year that are within suitable climate conditions for plant growth (i.e., suitable plant growing days) under different climate projections (see Methods; data used are described in S1–S2 Tables). We first estimated climatic thresholds (i.e., for temperature, soil moisture, solar radiation, and the interactions of these three factors) within which 95% of the terrestrial vegetative matter in the world is produced (Moderate Resolution Imaging Spectroradiometer [MODIS] Net Primary Production [NPP] from 2004–2013; S2 Table; see Methods; Fig 1). We then used daily climate projections (from the Coupled Model Intercomparison Project Phase 5 [CMIP5]) under strong (i.e., representative concentration pathway [RCP] 2.6), moderate (i.e., RCP 4.5), and business-as-usual (i.e., RCP 8.5) mitigation scenarios to quantify the number of days in a given year that fall within climate thresholds for plant growth. We analyzed each climate variable independently as well as their interactions. We describe results based on multimodel averages because they are more accurate at predicting observed suitable growing days than most models alone (results for precision and accuracy are shown in S2–S3 Figs).

**Fig 2:**
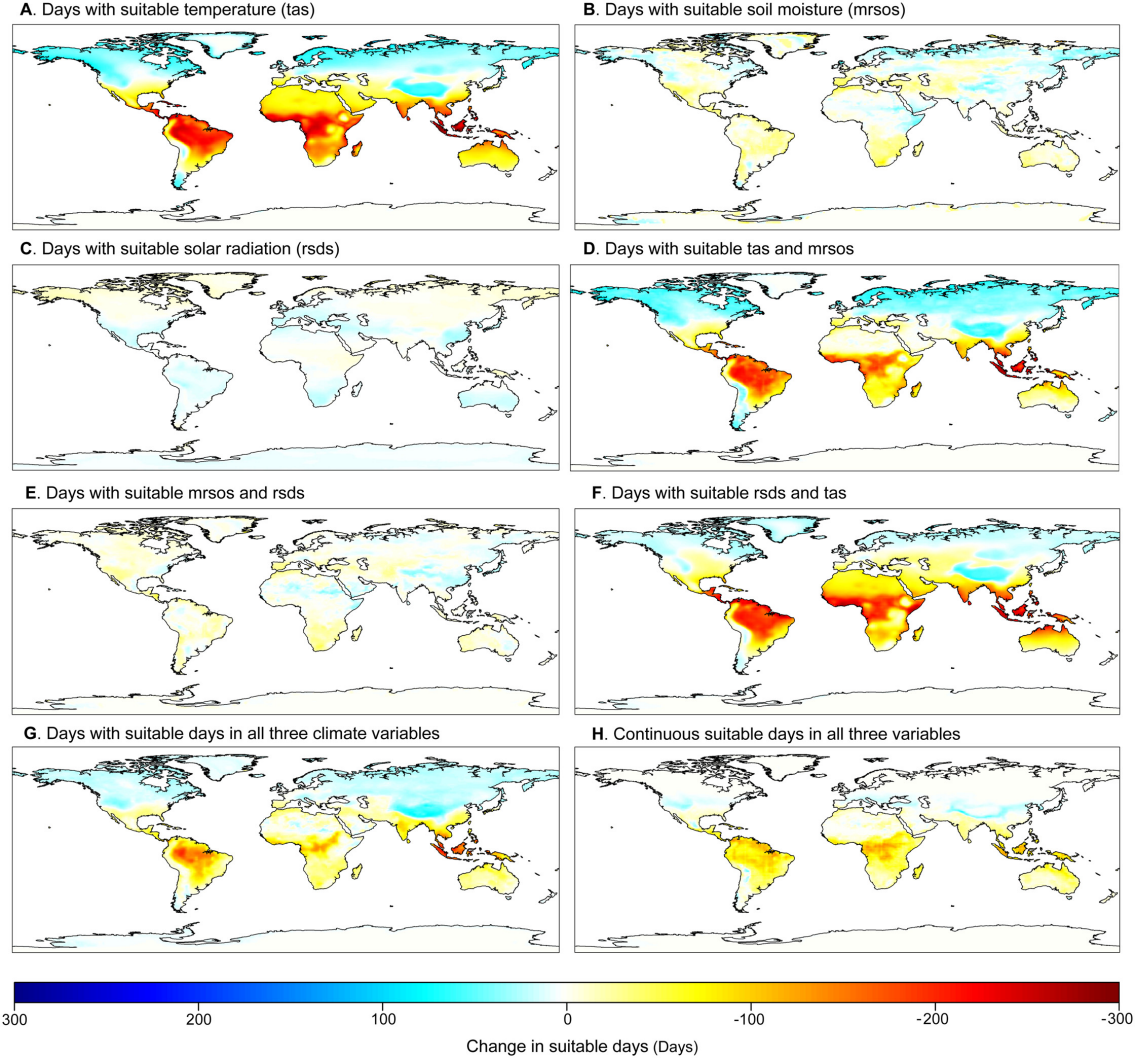
Spatial changes in projected suitable days for plant growth. Changes between future (i.e., the average from 2091 to 2100) and contemporary (i.e., the average from 1996 to 2005) number of days with suitable climatic conditions for plant growth under RCP 8.5 (results for all RCPs shown in S5–S7 Figs; data are provided in S2 Data). The map outline was obtained from the Central Intelligence Agency (CIA) World DataBank (https://www.evl.uic.edu/pape/data/WDB/).

**Fig 3:**
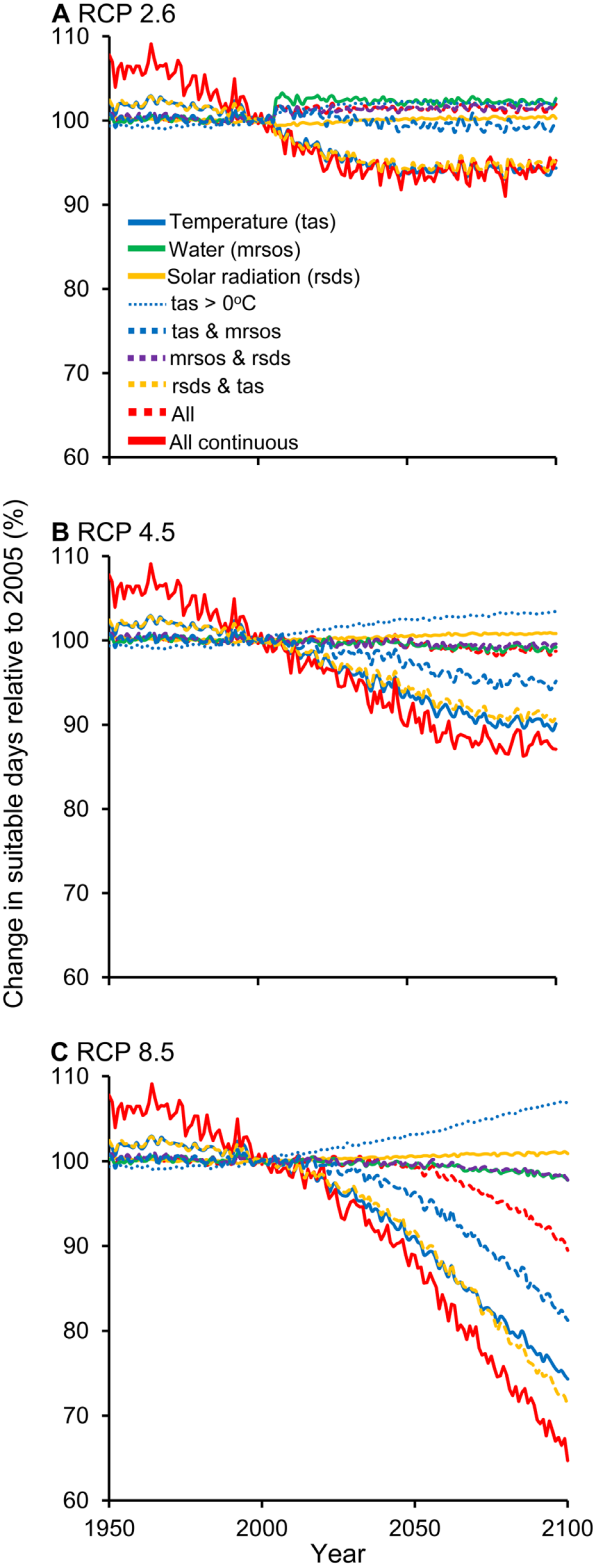
Global average changes in projected suitable days for plant growth. These plots illustrate the global average number of suitable plant growing days relative to contemporary values. Data are provided in S3 Data.

When we analyzed the limiting roles of temperature, soil moisture, and solar radiation independently, global average trends masked regional differences in the gains and losses of suitable plant growing days. As expected, we found that at mid- and high latitudes, projected warming will reduce the number of days below freezing, resulting in more suitable growing days (the average global number of days above freezing will increase by 2%, 5%, and 7% under RCP 2.6, RCP 4.5, and RCP 8.5, respectively; Fig 2A, S5A–S5D Fig, S6A–S6C Fig) [35]. However, we also found that warming will more often exceed the upper thermal threshold for plant growth, which will decrease the number of suitable growing days, mainly in the tropics (Fig 2A, S5A–S5D Fig, S6D–S6F Fig, see also [35]). By 2100, the decreasing number of suitable growing days in the tropics will offset optimistic projections at mid- and high latitudes, resulting in minimal changes in the global average number of suitable days under RCP 2.6 and RCP 4.5 but a ~26% reduction in the number of suitable growing days under RCP 8.5 (solid blue lines in Fig 3). For soil moisture and solar radiation, regional differences in the number of suitable plant growing days averaged out globally under all scenarios (solid green and yellow lines in Fig 3). Notably, projected changes in soil moisture (Fig 2B) and solar radiation (Fig 2C) showed contrasting spatial patterns. Areas that gained suitable days because of water availability also lost days because of solar radiation, and vice versa; this could be explained by coupled dynamics between rainfall and cloud cover [3].

Plant growth is strongly mediated by the extent to which multiple interacting climate variables remain within suitable conditions. When looking at the interaction between temperature and solar radiation, we found that the number of suitable plant growing days will decline more so than either variable independently (5%, 9%, and 29% under RCP 2.6, RCP 4.5, and RCP 8.5, respectively; dashed yellow lines in Fig 3). This steeper decline is driven mainly by patterns at high latitudes, where gains in suitable plant growing days due to higher temperatures are offset by the fact that those places remain limited by light (compare the intensity of blue colors in Fig 2A and 2F). In contrast, the interaction between temperature and soil moisture resulted in a smaller reduction in suitable plant growing days than the losses due solely to temperature (0%, 5%, and 19% under RCP 2.6, RCP 4.5, and RCP 8.5, respectively; dashed blue lines in Fig 3). This smaller decline is driven mainly by patterns in arid regions (e.g., northern Africa, Australia, and the Middle East), where losses in suitable plant growing days due to higher temperatures are reduced because those locations are already limited by water availability (compare yellow- and white-colored areas in Fig 2A and 2D). Changes in suitable plant growing days due to the interaction between solar radiation and soil moisture were minimal (-2%, 0%, and 2% under RCP 2.6, RCP 4.5, and RCP 8.5, respectively; dashed purple lines in Fig 3), although there was considerable spatial variability (Fig 2E) due to the coupling between rainfall and cloud cover. When looking at the interaction among all three climate variables, we found that the global average number of suitable days still decreased under RCP 8.5 but less so than when temperature was considered alone or in interaction with solar radiation or soil moisture (-2%, 1%, and 11% under RCP 2.6, RCP 4.5, and RCP 8.5, respectively; dashed red lines in Fig 3). Gains and losses in suitable plant growing days due to projected temperature changes alone are lessened because some regions are already limited by either solar radiation (reducing gains at high latitudes) or water availability (reducing losses in arid regions). However, there is still an overall loss in suitable plant growing days, with some regions facing unsuitable conditions for multiple reasons. In addition to fewer plant growing days, unsuitable plant climate conditions will occur sporadically throughout the year, as indicated by our metric of continuous suitable plant growing days. We found that the longest uninterrupted number of days when all three climate variables remained within suitable climate ranges reduced considerably under RCP 4.5 and RCP 8.5 (5%, 13%, and 35% under RCP 2.6, RCP 4.5, and RCP 8.5, respectively; solid red lines in Fig 3).

While some areas at high latitudes (most noticeably in Russia, China, and Canada) will gain days with suitable conditions in all three climate variables (Fig 2G, S5 Fig), many other areas will actually become limited by multiple climatic variables. For example, areas across the Sahel that are already limited by water availability will become increasingly limited by high temperatures by 2100 (Fig 2). These results highlight the risk for synergistic responses and concerns over biological and societal adaptations given the suite of physiological traits and social capacity needed to cope simultaneously with future changes in several climate variables. Reductions in the number of days with suitable climate conditions for plant growth also underscore an internal discrepancy of Earth System Models: while these models project dramatic enhancements of NPP [5,20,36], our results show multiple climate variables becoming limiting for plant growth, particularly in tropical areas, which could result in considerable reductions in future NPP (S4 Fig). This discrepancy likely reflects an overemphasis of CO_2_ fertilization in modeling NPP while failing to account for the limiting roles of other climatic variables and disturbances [5,22,36]. Furthermore, reductions in plant growth due to unsuitable growing days could lead to feedbacks whereby climate change is even more extreme, leading to even less suitable conditions for plant growth. The fact that unsuitable climatic conditions will occur more sporadically throughout the year highlights the potential for extreme events (e.g., heat waves or drought) to truncate the growing period, which may impair plant growth and even cause mortality [5,21,37]. Reichstein et al. [5] recently concluded that “climate extremes…can lead to a decrease in regional ecosystem carbon stocks and therefore have the potential to negate an expected increase in terrestrial carbon uptake,” further highlighting an important research area for improvement of Earth System Models.

**Fig 4:**
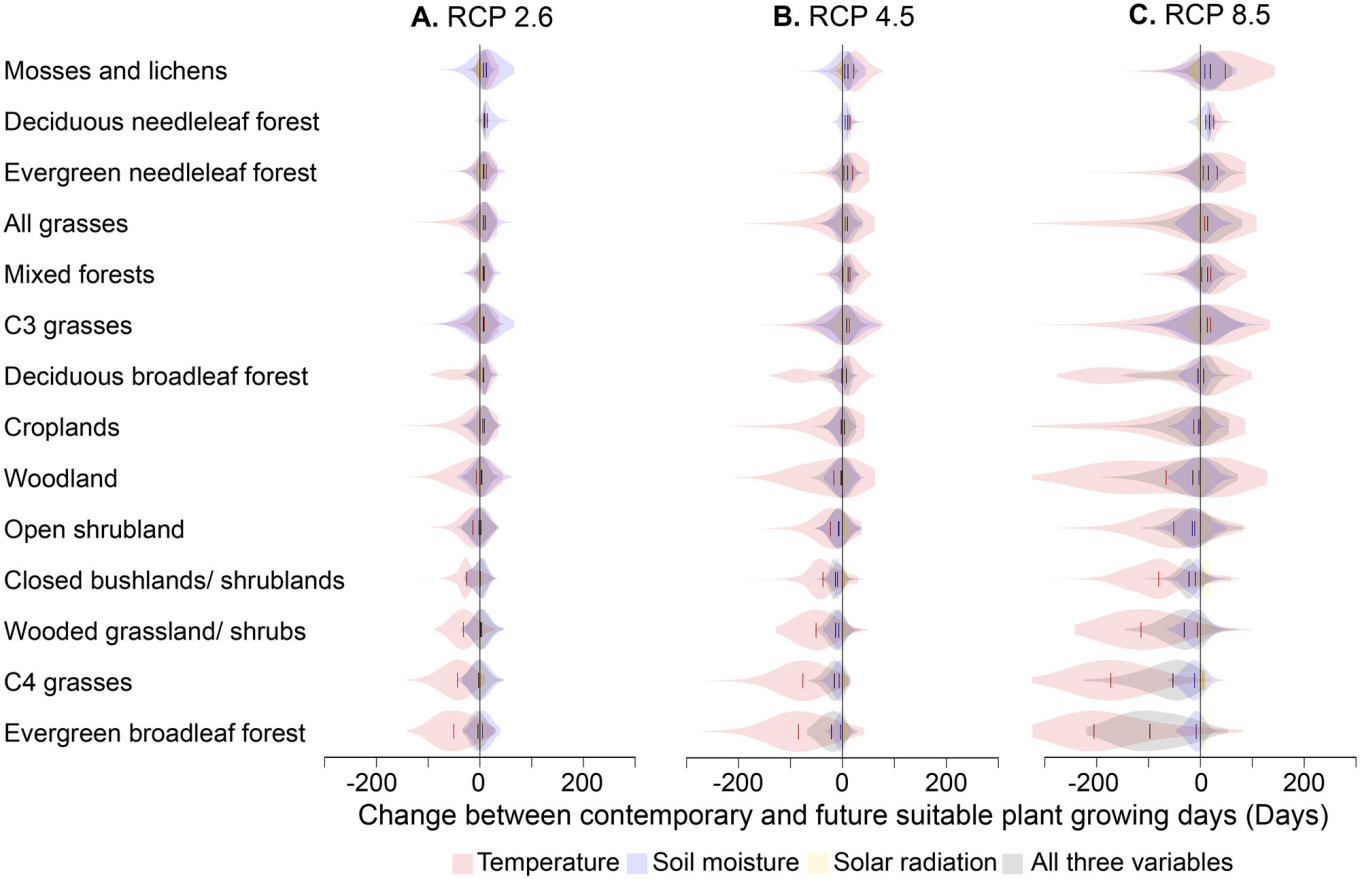
Biological exposure to projected changes in suitable plant growing days. Violin plots show frequency distributions of projected change between future and contemporary suitable plant growing days for all areas covered by each ecosystem; vertical colored lines indicate global median change for the given ecosystems. These plots are simply the overlay of our plant suitable days (data are provided in S2 Data) for areas of different land uses: (http://webmap.ornl.gov/wcsdown/wcsdown.jsp?dg_id=10006_1, http://webmap.ornl.gov/wcsdown/wcsdown.jsp?dg_id=20042_6, and http://webmap.ornl.gov/wcsdown/wcsdown.jsp?dg_id=20042_8).

Most of the world’s ecosystems and cultivated areas will be negatively affected by changes in the number of suitable growing days if climate change continues, possibly triggering climate feedbacks. Tropical ecosystems in particular (e.g., broadleaf evergreen forests; Fig 4) will lose suitable growing days due to temperatures exceeding the upper limit of the thermal range in combination with water failing to meet plant growth requirements. By 2100, for example, broadleaf evergreen forests will lose about 3 wk of suitable growing days under RCP 2.6 (Fig 4A) but lose nearly 3 mo under RCP 8.5 (Fig 4C). Prolonged unsuitable climatic conditions can prevent development of tropical forests [37] and result in tree die-offs, either directly from intolerance to altered climate conditions or indirectly through increased vulnerability to infestations by insects and pathogens [1,2,21]. In turn, such increased tree mortality can trigger ecological responses, including changes in plant community composition (e.g., from sensitive to less-sensitive species) and range contractions or expansions [2]. Unsuitable climate conditions can lead to increased plant respiration, potentially turning forests into carbon sources rather than carbon sinks [4,5]. At the same time, fewer freezing days at higher latitudes could potentially accelerate carbon releases through microbial decomposition [38,39], and this excess carbon might not be sequestered by plants, as higher latitudes will remain limited by insufficient solar radiation (S6G–S6I Fig). Finally, the impacts of climate change on plant growth could alter ecological interactions among species with potential cascading effects on food webs; integrating changes in suitable plant growing days and NPP within recently developed General Ecosystem Models [40] could provide some insights into the magnitude of these changes.

**Fig 5:**
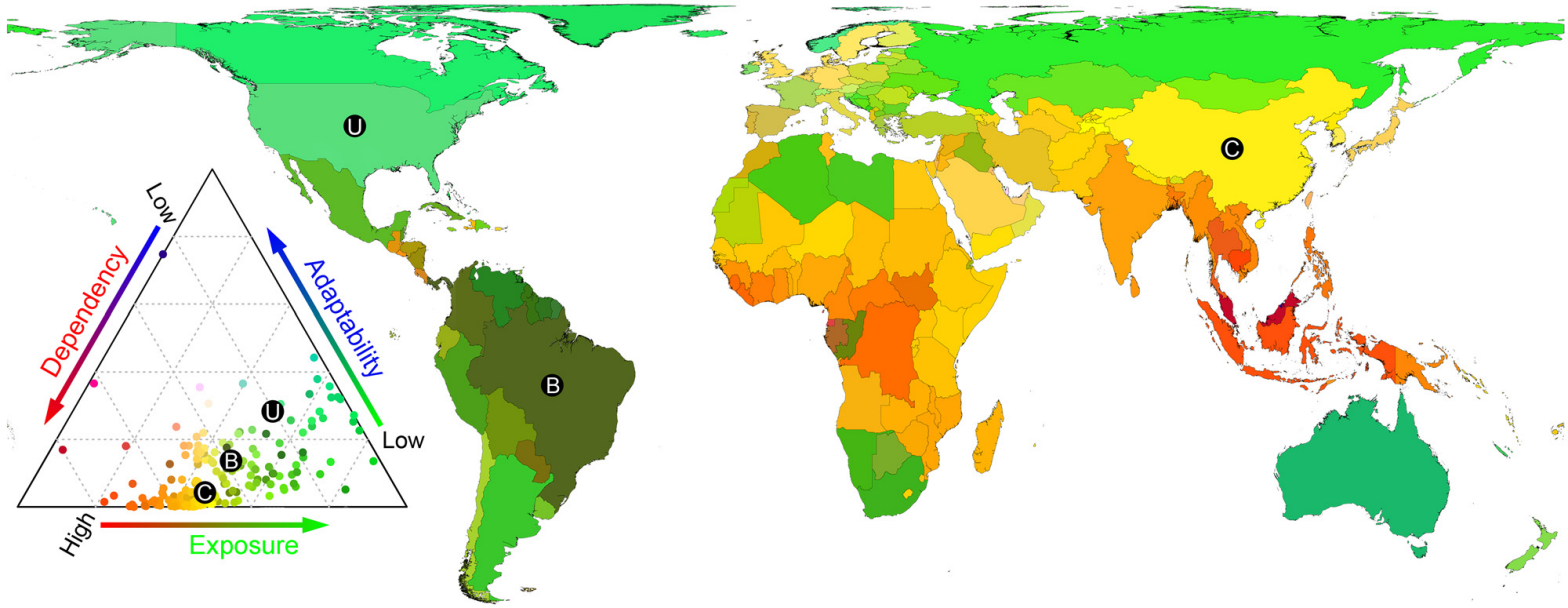
Human vulnerability to projected changes in suitable plant growing days. Human vulnerability is quantified as the combined effect of dependency, exposure, and adaptability, which are displayed along a red-green-blue gradient (colors in the triangle correspond to colors in the map). Points in the triangle represent each of the 194 countries analyzed, with the positions of United States (U), China (C), and Brazil (B) indicated for reference. The map outline was obtained from the CIA World DataBank (S2 Table). Data are provided in S4 Data.

Losses in suitable plant growing days can translate into losses of food, fiber, fuel, and associated jobs and revenue, with potentially negative effects in countries with high reliance on those goods and services, particularly those with minimal capacity to adapt. Here, we assessed human vulnerability to changes in the number of suitable plant growing days by using a common method that distinguishes populations depending on their (i) “exposure” to environmental change, (ii) “dependency” on potentially impacted goods and services, and (iii) social “adaptability” [41–44]. We used changes in suitable plant growing days (i.e., between contemporary and 2100, Fig 2G) as our metric of exposure and collected agriculture-related and economic data to quantify dependency and adaptability (see Methods). Under RCP 2.6, no country will experience high losses or high gains in suitable plant growing days (i.e., reductions or gains greater than 30% of the current suitable growing period, S3 Table). However, human vulnerability will be much greater under RCP 8.5. If climate change were to continue under this scenario, ~3,400 million people will live in countries facing reductions of 30% or more suitable plant growing days; of those people, ~2,900 million are highly dependent on plant-related goods and services, and ~2,100 million of those are in low-income countries (S3 Table). A few countries in the Americas and all countries in Oceania, Asia, and Africa, with the exception of Australia, New Zealand, Russia, South Africa, Namibia, Algeria, and Libya, are highly vulnerable to reductions in plant growing days (Fig 5). Under RCP 8.5, only ~270 million people live in countries projected to experience medium to high gains (i.e., greater than 10%) in the number of suitable plant growing days (e.g., Iceland, Norway, Sweden). Vulnerability for all countries is shown in Fig 5, S3 Table, and S2 Data.

**Fig 6:**
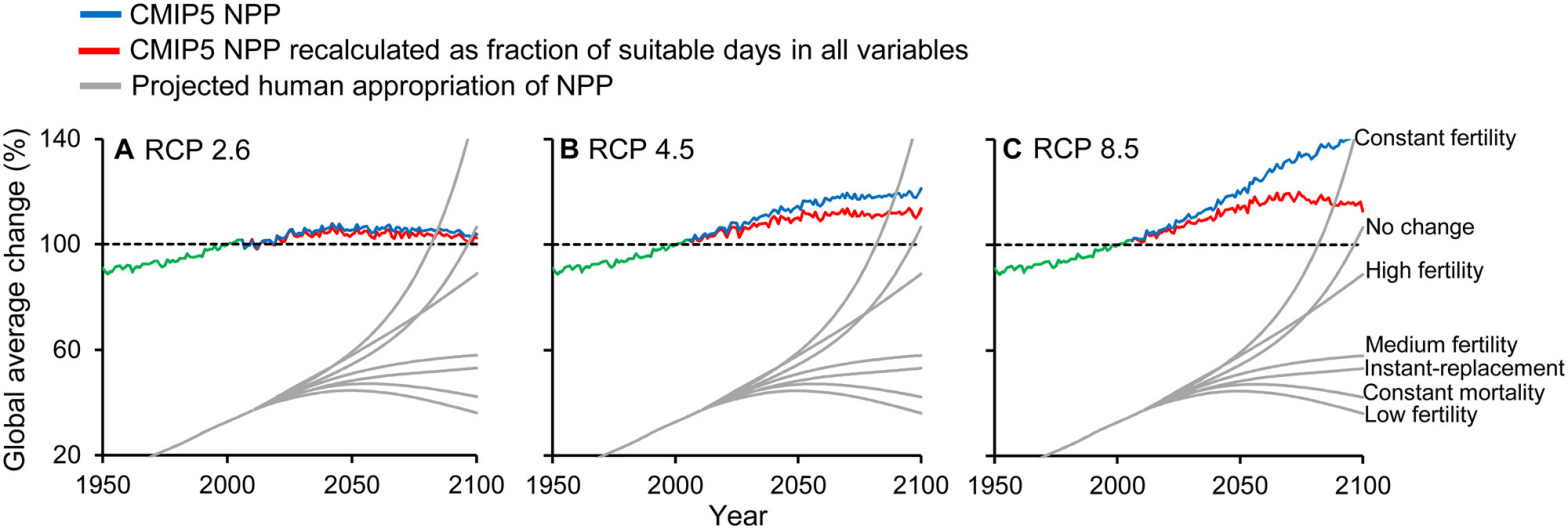
Projected changes in NPP under different scenarios of emissions and human consumption of terrestrial NPP. Plots A–C show the global average change in NPP under different scenarios before (blue lines) and after (red lines) accounting for unsuitable plant growing days. Grey lines indicate the projected global human appropriation of terrestrial NPP (i.e., modern per capita appropriation of NPP multiplied by human population projections under different scenarios). Additional details are shown in S4 Fig. Data are provided in S5 Data.

Our study adds to the understanding of projected changes in climate suitability for plant growth, highlighting where ecosystems and human populations could be more vulnerable to such changes. Although our study confirms a benefit of ongoing climate change on plant growing conditions at higher latitudes because of fewer freezing days, this considerably underestimates the full extent of consequences of projected climate changes, particularly under business-as-usual projections. Consideration of an upper thermal limit and interactions with plant growth thresholds in additional climatic variables resulted in the opposite trend: global decreases in the number of suitable plant growing days by 2100 (Fig 2). The unprecedented rate and number of climate variables becoming limiting for plant growth could challenge the capacity of species to adapt, with the potential to negatively impact terrestrial ecosystems and trigger climate feedbacks. Potential reductions in plant growth associated with fewer plant growing days are particularly worrisome given that the largest impacts are expected to affect the poorest and most agriculturally dependent countries in the world (Fig 5). These effects will be further exacerbated by increasing human appropriation of NPP associated with human population growth (Fig 6, S4 Fig). On a positive note, our study also indicated that projected changes in suitable plant growing days are minimal under RCP 2.6, underscoring the importance of reducing emissions to avoid such disproportionate impacts on ecosystems and people.

## Methods

### Quantifying Plant Growth Climatic Thresholds

We used the rate at which terrestrial vegetative matter is produced (NPP) as a proxy for plant growth. Derived values of NPP were obtained from 8-d averaged MODIS data (the finest temporal resolution available; data source in S2 Table). MODIS NPP data are modelled using remotely sensed satellite data and have been cross-validated by other studies [45]. To estimate climate thresholds for plant growth, we overlaid 8-d maps of derived NPP onto 8-d maps of observed temperature (i.e., near-surface air temperature), water availability (using soil moisture in upper 10 cm of the soil column as proxy), and solar radiation (i.e., surface downwelling shortwave radiation) (sources provided in S2 Table). This allowed us to calculate the total amount of 8-d NPP produced along gradients of each of the three climate variables and their interactions. We defined NPP climatic thresholds as the boundaries that surround the climatic conditions under which 95% of the world’s NPP occurs for each variable (Fig 1A–1C) and their interactions (Fig 1D–1G), for each year between 2004 and 2013. For our analysis, we used the boundaries encompassing all of the yearly boundaries (Fig 1) and define suitable growing days as those days in which projected climatic conditions fall within that multiyear boundary. While some plants grow under extreme conditions, relatively little NPP occurs in these primarily cold and arid places (as noted by the steep declines of NPP along climatic variables in Fig 1); using more than 95% of global NPP to include these extremist plants will considerably broaden the climate thresholds and overestimate global suitability for the majority of plant growth. An upper threshold for radiation was rarely exceeded (S7B Fig), but we retained it to maintain consistency with the analysis of other climatic variables. To compare global thresholds to ecosystem-specific thresholds, we repeated the above approach using NPP within cells that overlap each of 14 land-cover types (based on satellite-derived maps of dominant ecosystem type; S8 Fig, data sources provided in S2 Table under “Land use data”). We used global thresholds to calculate suitable plant growing days, as they encompassed the bulk productivity of most ecosystems (S8 Fig). However, some ecosystems that already frequently experience extreme conditions surpassed global thresholds (e.g., semidesert wooded grassland/shrubs, S8 Fig), suggesting that these ecosystems could better cope with future climate projections. Climatic thresholds were also very similar if they were weighted by the area where climatic conditions occur (S1 and S8 Figs). All data sources are listed in S2 Table.

### Calculating Suitable Plant Growing Days

To estimate the number of suitable days for plant growth each year, we counted the total or consecutive number of days in a year in which climatic conditions (i.e., temperature, soil moisture, solar radiation, and the interactions of these three variables) fall within the global thresholds for plant growth. We obtained daily projections of temperature, soil moisture, and solar radiation from recent Earth System Models developed as part of the Coupled Model Intercomparison Project Phase 5 to the Fifth Assessment Report of the Intergovernmental Panel on Climate Change (S1 Table). Daily projections run from 1950 to 2005 simulating anthropogenic and natural climate forcing (i.e., “historical” experiment) and from 2006 to 2100 under three alternative representative concentration pathways: RCP 2.6, RCP 4.5, and RCP 8.5. CO_2_ concentrations will reach ~400, ~530, and ~930 ppm by 2100, under RCP 2.6, RCP 4.5, and RCP 8.5, respectively. As of November 2014, there were 14 Earth System Models from 12 centers in eight countries that modeled temperature, soil moisture, and solar radiation at a daily resolution for at least one of the three RCPs (S1 Table) (Note: all Earth System Models that we used include feedbacks of plant production on water balance). In total, for all variables and projections, we processed ~1.8 million daily global maps. We quantified the number of suitable plant growing days independently for each model and averaged the results to estimate the multimodel average. Changes in the number of suitable plant growing days (Fig 2) were calculated by subtracting contemporary (1996 to 2005) from future averages (2091 to 2100); decadal averages were chosen to minimize aliasing by interannual variability. To assess exposure of different terrestrial ecosystems to projected changes in climate suitability (Fig 4), we calculated the mean and frequency distribution of changes in suitable plant growing days (Fig 2A–2C and 2G) for cells dominated by each of 14 land-cover types. All data sources are indicated in S2 Table.

### Assessing Human Vulnerability

“Vulnerability” was assessed in the traditional sense of determining human “exposure” to environmental change, “dependency” in terms of food, jobs, and revenue at stake, and “adaptability” in terms of wealth, assuming that richer countries will have more capacity to respond [41–43]. “Exposure” was quantified as changes in climate suitability for plant growth categorized for each country as follows: “high loss” for countries experiencing reductions in suitable plant growing days in excess of 30%, “medium loss” for countries experiencing losses of 30% to 10%, “no change” for countries that gain or lose up to 10%, “medium gain” for countries gaining 10% to 30%, and “high gain” for countries gaining in excess of 30% more days. “Dependency” was quantified by adding three proportional metrics for each country: percentage of gross domestic product contributed by agricultural revenue, percentage of the workforce in the agricultural sector, and percentage of NPP appropriated by people (from food, paper, wood, meat, fiber, and animal by-products) [14]. Countries were categorized as having “low,” “medium,” or “high” dependency if their cumulative percentages in those three goods and services ranged from 0% to 33%, >33% to 66%, or >66%, respectively. Finally, “adaptability” was quantified as per capita gross domestic product, under the assumption that richer countries will have greater access to a wider range of adaptive strategies. For the purpose of classification, we used the World Bank categorization of low-, medium-, and high-income countries depending on whether annual per capita gross domestic product was less than US$4,000, between US$4,000 and US$12,000, or greater than US$12,000, respectively. All data sources are shown in S2 Table.

### General Considerations and Caveats

#### Variability in thresholds

To project global plant growth suitability, we used thresholds broad enough to encompass the current sensitivities of most plants (S8 Fig) and kept them static for comparison, but we recognize that those thresholds can change. Thresholds could change either at the species level (through genetic adaptation, but see [46]) or at the community level (through replacement of species with those that are more tolerant today [2] or those that have greater adaptive capacity [47]). It would be expected that more diverse ecosystems will have greater capacity to deal with projected unsuitable climates compared to monoculture systems (i.e., more diverse ecosystems should have a greater variety of thresholds [7]). This highlights the vulnerability of many agricultural systems and associated human vulnerability to future climatic changes, as necessary adjustments to farming practices (e.g., using more tolerant crop varieties, irrigation, etc.) are likely to be costly and some of the most extreme reductions in plant growing days are expected in tropical countries with limited economic capacity (Figs 2 and 5). Interactions among CO_2_ and climatic variables could also broaden or narrow modern thresholds. For instance, elevated CO_2_ is known to increase resistance to drought by plants closing their stoma [48,49]. However, under warming conditions the closing of the stoma may induce overheating (by preventing transpiration) and/or if sustained could decrease carbon fixation [50,51]. Likewise, the temperature ranges over which elevated CO_2_ enhances plant growth are strongly mediated by water availability [49]. Our paper described climate suitability for plant production overall, but we also provide a web-user interface that allows for repetition of our analysis with tolerance thresholds related to different adaptation scenarios and species- and/or ecosystem-specific thresholds (http://128.171.126.15/growingdays/index.html).

#### Correlative nature of our approach

Our analysis uses the modern distribution of where plants grow and assumes that climatic conditions at those locations are suitable. Although this is a correlative approach, it provides important relative insights into how plant growth could be affected by alternative future climates.

#### Additional global sources of NPP

It should be noted that a major source of the world’s productivity includes freshwater and marine plants, which could not be incorporated into the scope of this study because they are not limited by the same climatic conditions (e.g., soil moisture) as terrestrial NPP. Our approach could be replicated for those systems using the climatic variables that limit their productivity. This would represent another interesting study.

#### Population projections and human vulnerability

Our calculations for the number of people vulnerable to projected changes in suitable plant growing days were based on current population numbers (as of 2012), but populations are projected to increase to 9,600–12,300 million people by 2100 [52]. These projections suggest that the number of people vulnerable to projected changes in suitable plant growing days will be higher than indicated in this paper. Human vulnerability could be further exacerbated because projected increases in human population are likely to result in a higher demand for diminishing plant-associated resources (Fig 6, S4 Fig, [29]).

## Supporting Information

S1 Data Data for Fig 1. (XLSX)

S2 Data Data for Fig 2. (XLS)

S3 Data Data for Fig 3. (XLSX)

S4 Data Data for Fig 5. (XLS)

S5 Data Data for Fig 6. (XLSX)

S6 Data Data for S2 Fig. (XLSX)

S7 Data Data for S3 Fig. (XLSX)

S8 Data Data for S6 Fig. (XLS)

S9 Data Data for S7 Fig. (XLSX)

S1 Fig Comparison of climatic ranges for plant growth with and without weighting by area. (DOCX)

S2 Fig Robustness of Earth System Models in estimating suitable days for plant growth. (DOCX)

S3 Fig Multimodel uncertainty. (DOCX)

S4 Fig Projected changes in NPP from CMIP5 Earth System Models before and after accounting for unsuitable plant growing days and their comparison with projected human appropriation of NPP. (DOCX)

S5 Fig Projected changes in suitable days for plant growth. (DOCX)

S6 Fig Specific climatic thresholds limiting plant growth. (DOCX)

S7 Fig Temporal changes in the relative importance of limiting climatic variables for plant growth. (DOCX)

S8 Fig Climatic thresholds by ecosystems and land use types. (DOCX)

S1 Table Earth System Models analyzed. (DOCX)

S2 Table Data sources. (DOCX)

S3 Table Human vulnerability to changes in suitable days for plant growth. (DOCX)

## Abbreviations

CIA: Central Intelligence Agency
CMIP5: the Coupled Model Intercomparison Project Phase 5
NCEP: National Centers for Environmental Prediction
NPP: Net Primary Production
MODIS: Moderate Resolution Imaging Spectroradiometer
RCP: representative concentration pathway
