# Supplementary material for: Suitable Days for Plant Growth Disappear under Projected Climate Change: Potential Human and Biotic Vulnerability
Source: PLoS Biol. 2015 Jun 10;13(6):e1002167. doi: 10.1371/journal.pbio.1002167 (PMC4465630; doi:10.1371/journal.pbio.1002167)

**Fig. S1. Comparison of climatic ranges for plant growth with and without weighting by area.** For this analysis, patterns of NPP along climatic variables (blue lines) were reassessed (red lines) by dividing the amount of NPP occurring at each climatic condition by the global area where such climatic conditions occur. Data provided in S1 Data.

NPP data: http://neo.sci.gsfc.nasa.gov/view.php?datasetId=MOD17A2_E_PSN

Climate data: http://www.esrl.noaa.gov/psd/cgi-bin/db_search/DBSearch.pl?Dataset=NCEP+Reanalysis+Daily+Averages+Surface+Flux&group=0&submit=Search


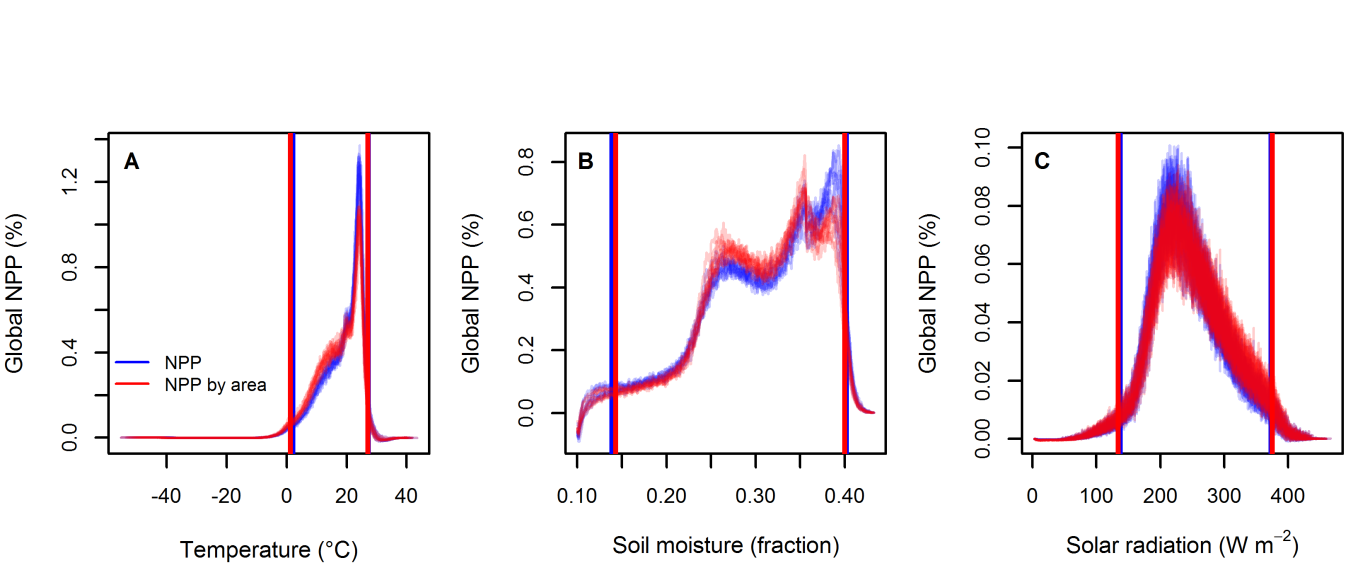

Supplement: S1 Fig — (DOCX) [file pbio.1002167.s010.docx]
