# Supplementary material for: Suitable Days for Plant Growth Disappear under Projected Climate Change: Potential Human and Biotic Vulnerability
Source: PLoS Biol. 2015 Jun 10;13(6):e1002167. doi: 10.1371/journal.pbio.1002167 (PMC4465630; doi:10.1371/journal.pbio.1002167)

**Fig. S2. Robustness of Earth System Models in estimating suitable days for plant growth**. For this analysis, we compared the number of suitable plant growing days estimated from observed data on temperature, solar radiation and soil moisture with the number of days estimated using climate projections from Earth System Models. Suitable growing days were estimated for each year from 1996 to 2005, averaged over that decade and compared in the plots below. The similitude between observed and projected suitable plant growing days is displayed here using normalized Taylor diagrams, which display three metrics of similitude concurrently: the correlation (curved axis), the ratio of the standard deviations (x- and y-axes) and the root mean squared error (blue arcs). Blue points indicate perfect fit, red points the multi-model average, and black points the comparison of each Earth System Model to actual observations. The closer a red or black point is to the blue point, the better the fit between actual and projected data. Data provided in S6 Data.


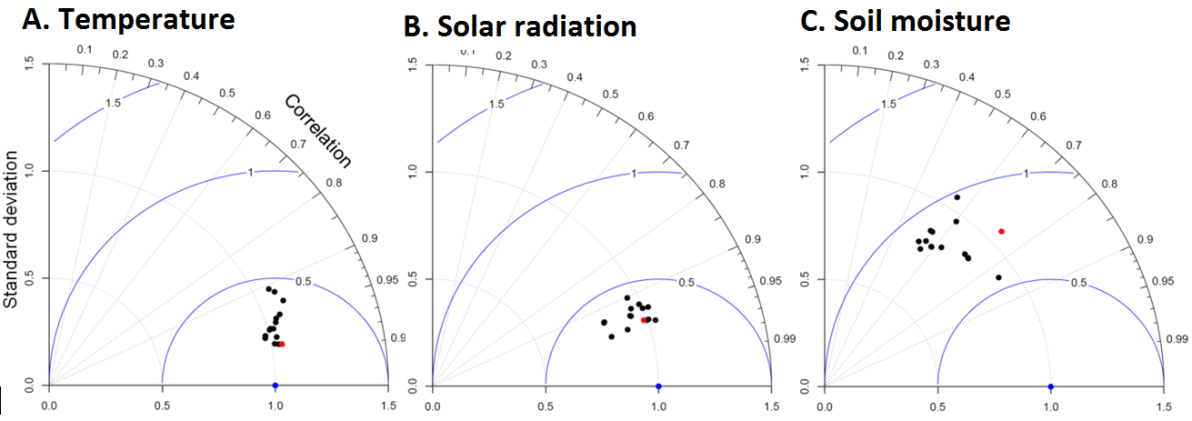

Supplement: S2 Fig — (DOCX) [file pbio.1002167.s011.docx]
