# Supplementary material for: Suitable Days for Plant Growth Disappear under Projected Climate Change: Potential Human and Biotic Vulnerability
Source: PLoS Biol. 2015 Jun 10;13(6):e1002167. doi: 10.1371/journal.pbio.1002167 (PMC4465630; doi:10.1371/journal.pbio.1002167)
Supplement: S3 Fig — (DOCX) [file pbio.1002167.s012.docx]

**Fig. S3. Multi-model uncertainty.** Global maps showing the multi-model projected change between future (i.e., average from 2091-2100) and contemporary (i.e., average from 1996-2005) suitable growing days under RCP 8.5 (maps on the left) in comparison to multi-model uncertainty (i.e., the variability in suitable growing days among the 14 CMIP5 models used; middle maps). Frequency histograms (on the right) show the fraction of global land area in which the projected change exceeds the multi-model uncertainty (positive numbers on x-axis) and vice-versa (negative numbers on the x-axis). Data for standard deviations provided in S7 Data. Data for absolute changes provided in S2 Data.

**
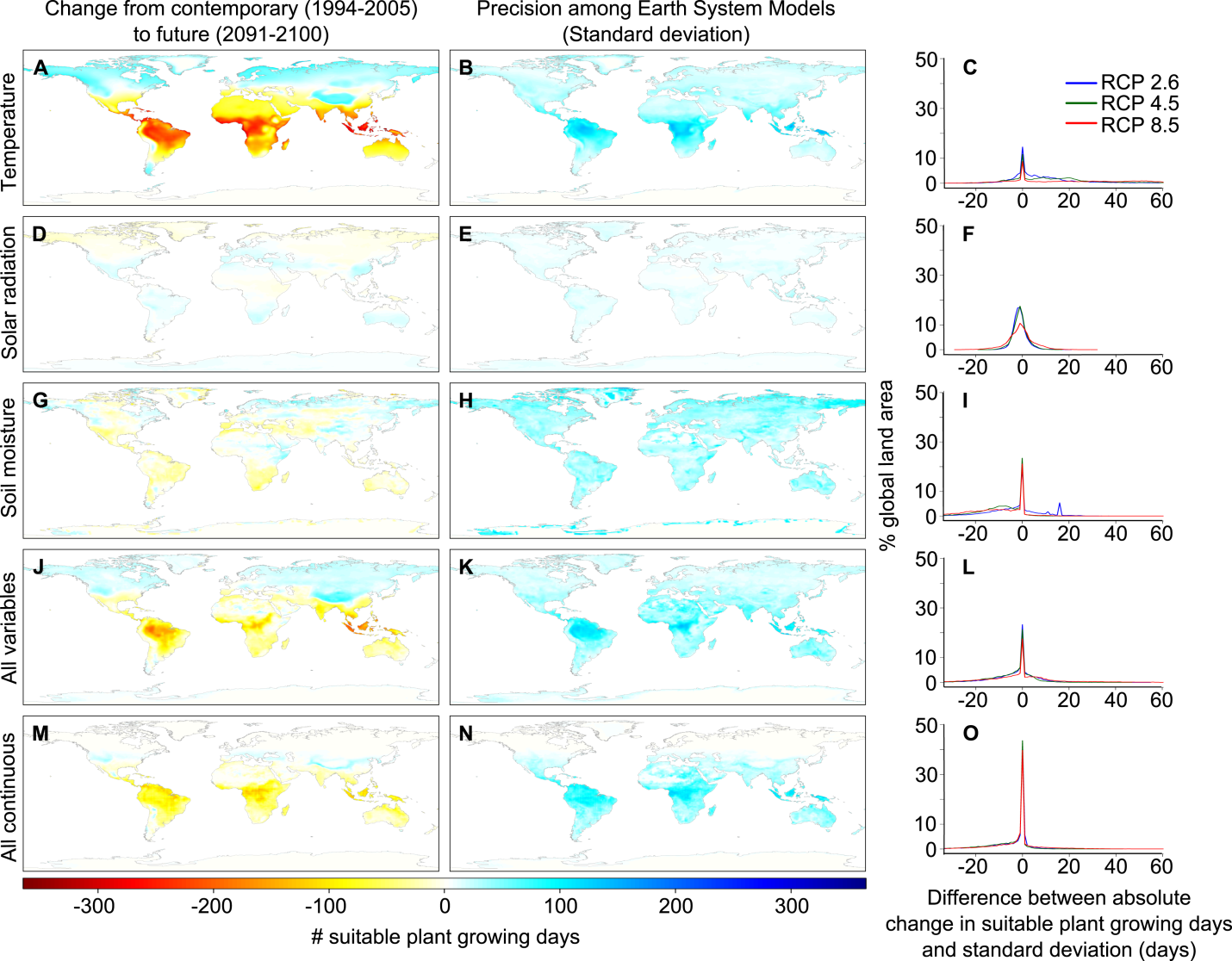
**
