# Supplementary material for: Suitable Days for Plant Growth Disappear under Projected Climate Change: Potential Human and Biotic Vulnerability
Source: PLoS Biol. 2015 Jun 10;13(6):e1002167. doi: 10.1371/journal.pbio.1002167 (PMC4465630; doi:10.1371/journal.pbio.1002167)

**Fig. S4. Projected changes in NPP from CMIP5 Earth System Models before and after accounting for unsuitable plant growing days and their comparison with projected human appropriation of NPP.** In these plots, change refers to the difference between contemporary (i.e., the average from years 1996 to 2005) and future (i.e., the average from 2091 to 2100) periods. Plots A-C show the global average change in NPP under different scenarios before (blue lines) and after (red lines) accounting for unsuitable plant growing days. To account for unsuitable plant growing days, monthly CMIP5 NPP was multiplied by the fraction of suitable days for each month (i.e., suitable days in month *i* in year *j* /days in month *i*) and the resulting monthly NPP was added for each year. These results were very similar if CMIP5 NPP was alternatively calculated relative to the number of suitable days in the contemporary period (i.e., suitable days in month *i* in year *j* / suitable days in month *i* in contemporary period). Grey lines indicate the projected global human consumption of terrestrial NPP calculated based on human per capita appropriation of NPP (i.e., as averaged from [[13](#_ENREF_13),[14](#_ENREF_14),[17](#_ENREF_17),[18](#_ENREF_18)], but see [[17](#_ENREF_17)]) multiplied by population projections from the United Nations World Population Prospects (http://esa.un.org/wpp/Excel-Data/population.htm). Maps (D-L) further show the spatial differences in NPP from CMIP5 models before (D-F) and after (G-I) recalculating NPP proportional to the number of suitable plant growing days in the future (J-L). Data provided in S5 Data.


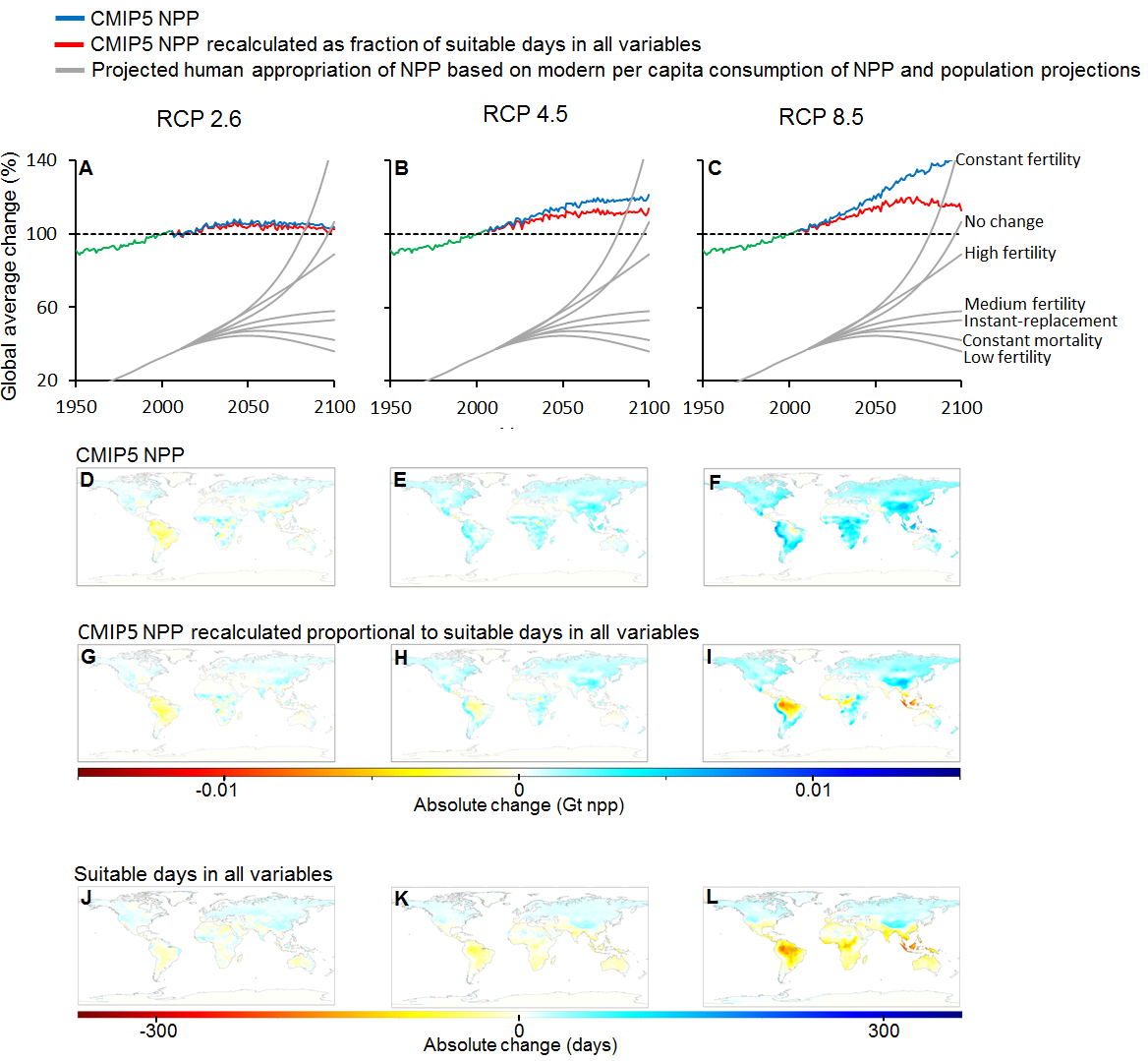

Supplement: S4 Fig — (DOCX) [file pbio.1002167.s013.docx]
