# Supplementary material for: Suitable Days for Plant Growth Disappear under Projected Climate Change: Potential Human and Biotic Vulnerability
Source: PLoS Biol. 2015 Jun 10;13(6):e1002167. doi: 10.1371/journal.pbio.1002167 (PMC4465630; doi:10.1371/journal.pbio.1002167)
Supplement: S5 Fig — (DOCX) [file pbio.1002167.s014.docx]

**Fig. S5. Projected changes in suitable days for plant growth.** Maps illustrate changes in number of suitable days for plant growth between contemporary and future time periods under different RCPs. The same as Fig. 2, which shows the results for just RCP 8.5. Data provided in S2 Data.

**
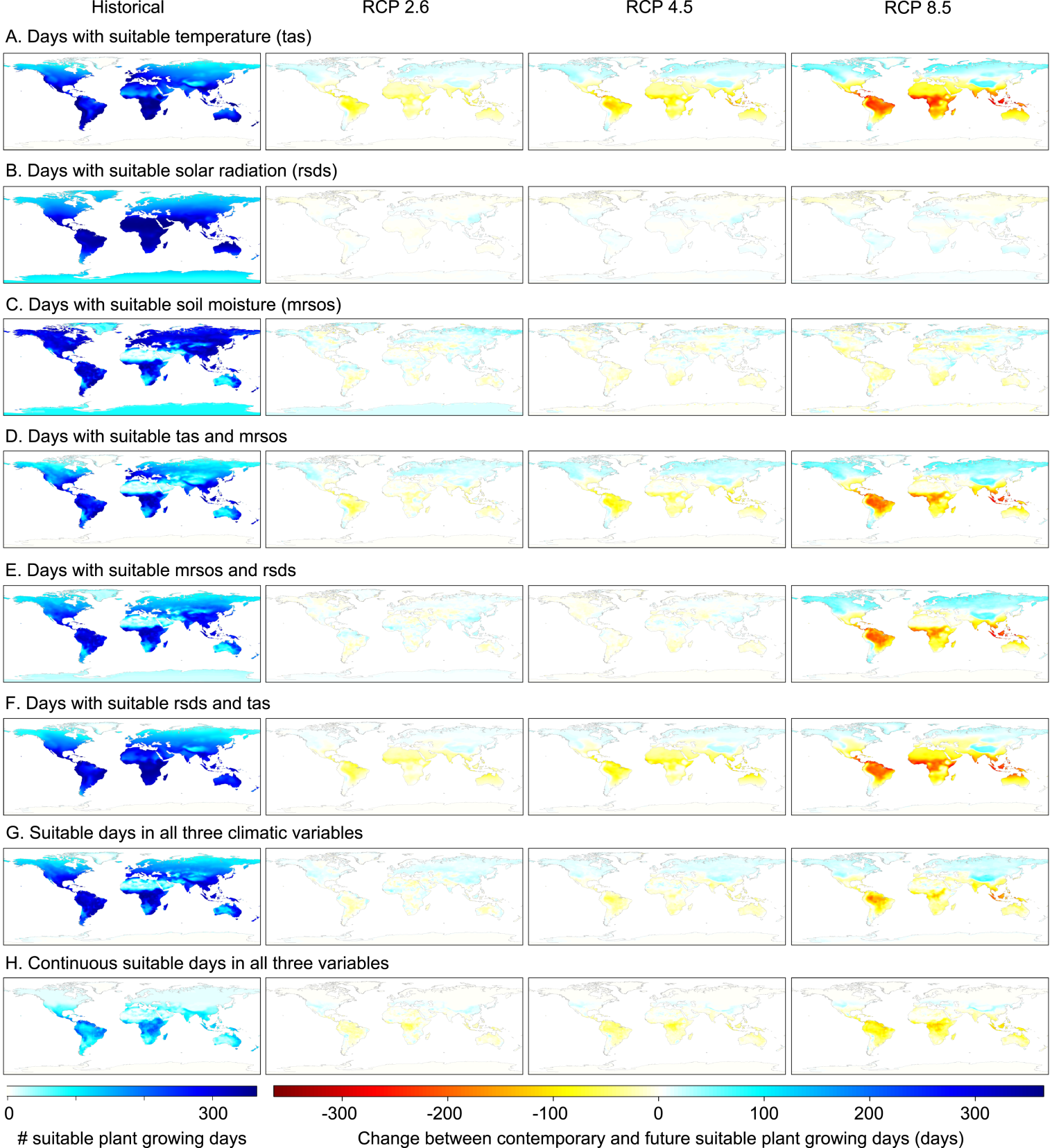
**
