# Supplementary material for: Suitable Days for Plant Growth Disappear under Projected Climate Change: Potential Human and Biotic Vulnerability
Source: PLoS Biol. 2015 Jun 10;13(6):e1002167. doi: 10.1371/journal.pbio.1002167 (PMC4465630; doi:10.1371/journal.pbio.1002167)
Supplement: S6 Fig — (DOCX) [file pbio.1002167.s015.docx]

**Fig. S6. Specific climatic thresholds limiting plant growth.** These maps illustrate changes in unsuitable plant growing days by whether the lower or upper limits of each climate variable is exceeded. Results are shown by contemporary (maps on the left) and future (middle maps) limiting conditions and their differences (maps on the right). Data for this figure are provided in S8 Data.

**
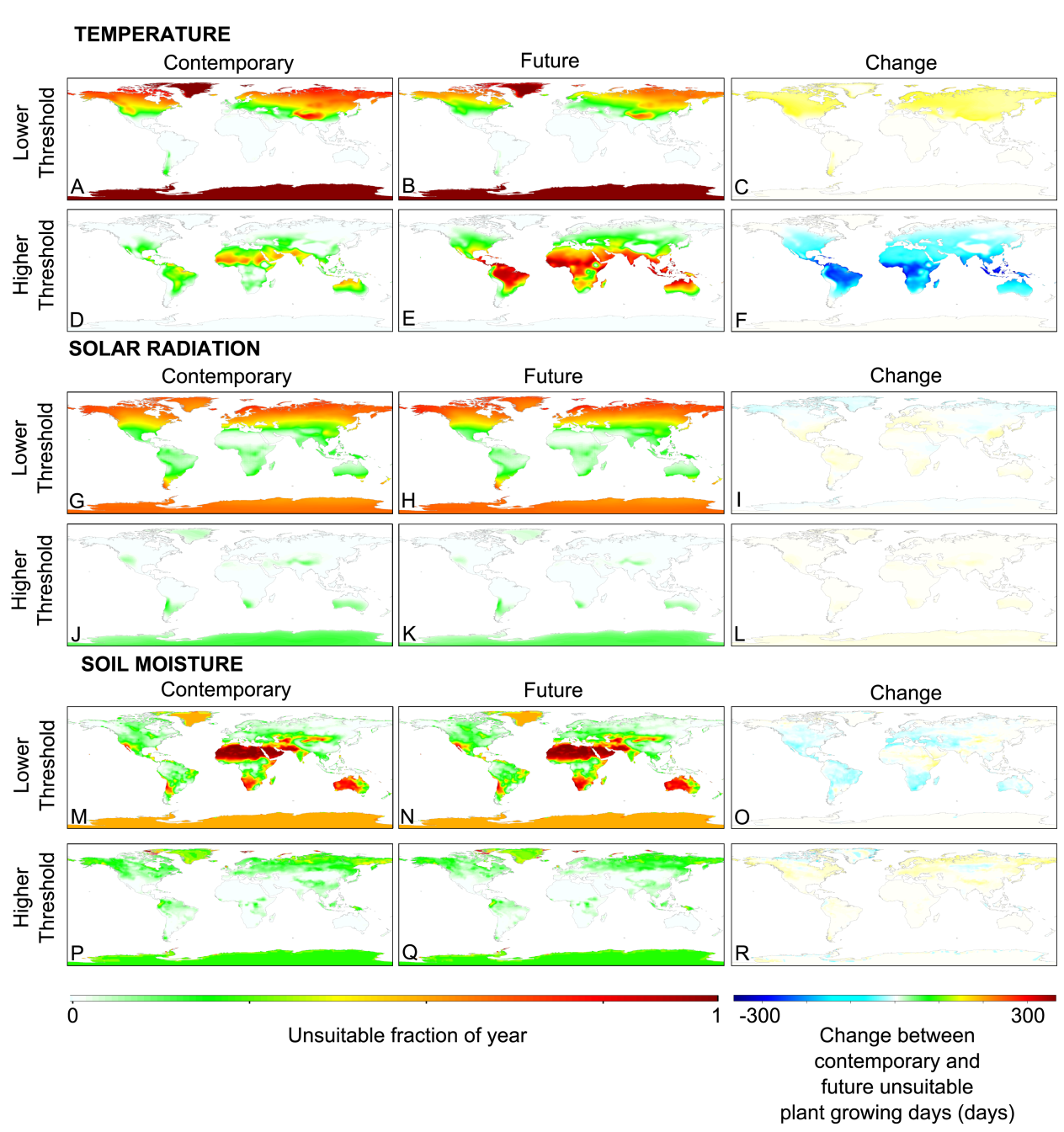
**
