# Supplementary material for: Suitable Days for Plant Growth Disappear under Projected Climate Change: Potential Human and Biotic Vulnerability
Source: PLoS Biol. 2015 Jun 10;13(6):e1002167. doi: 10.1371/journal.pbio.1002167 (PMC4465630; doi:10.1371/journal.pbio.1002167)

**Fig. S7. Temporal changes in the relative importance of limiting climatic variables for plant growth.** These plots indicate the global average number of days per year limited by climate variables exceeding lower (dashed lines) and upper (solid lines) climatic thresholds for plant growth. Under future climate change scenarios, low temperatures become less limiting to plant growth compared to an increased limiting role of high temperatures (A). Data provided in S9 Data.


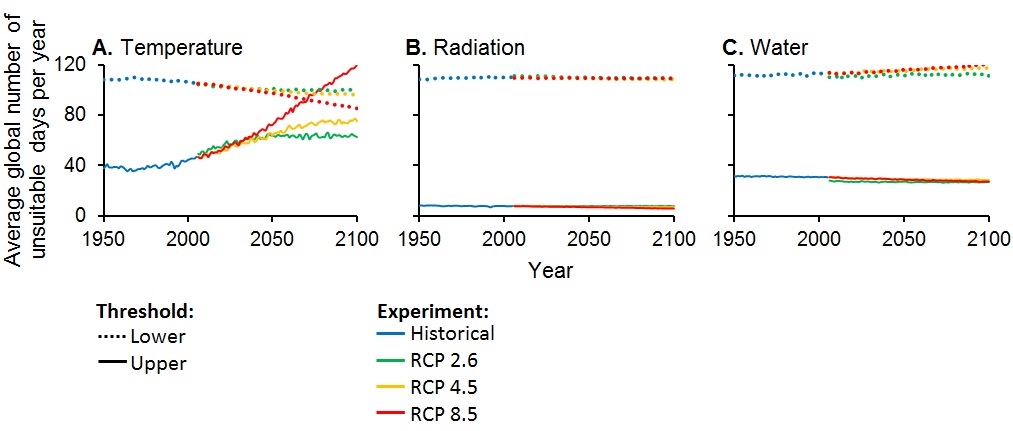

Supplement: S7 Fig — (DOCX) [file pbio.1002167.s016.docx]
