# Supplementary material for: Suitable Days for Plant Growth Disappear under Projected Climate Change: Potential Human and Biotic Vulnerability
Source: PLoS Biol. 2015 Jun 10;13(6):e1002167. doi: 10.1371/journal.pbio.1002167 (PMC4465630; doi:10.1371/journal.pbio.1002167)

**Fig. S8. Climatic thresholds by ecosystems and land use types.** Each plot represents the proportion of NPP produced along climatic gradients for each of 14 global land-cover types, relative to the maximum NPP produced at any given climatic condition. Grey x-axes indicate zero NPP. Blue and red vertical lines mark the thresholds under which 95% of the NPP in each land-cover type occur with and without weighting by area, respectively (this is the same as Fig. S1 but for each ecosystems and land use type). Thresholds were based on data from 2004-2013 and calculated using the same approach as the global climate thresholds for plant growth (i.e., Fig. 1A-C), and shown here as vertical dashed lines. These plots are the overlay of climatic conditions on different land use type (data sources provided in Table S2).


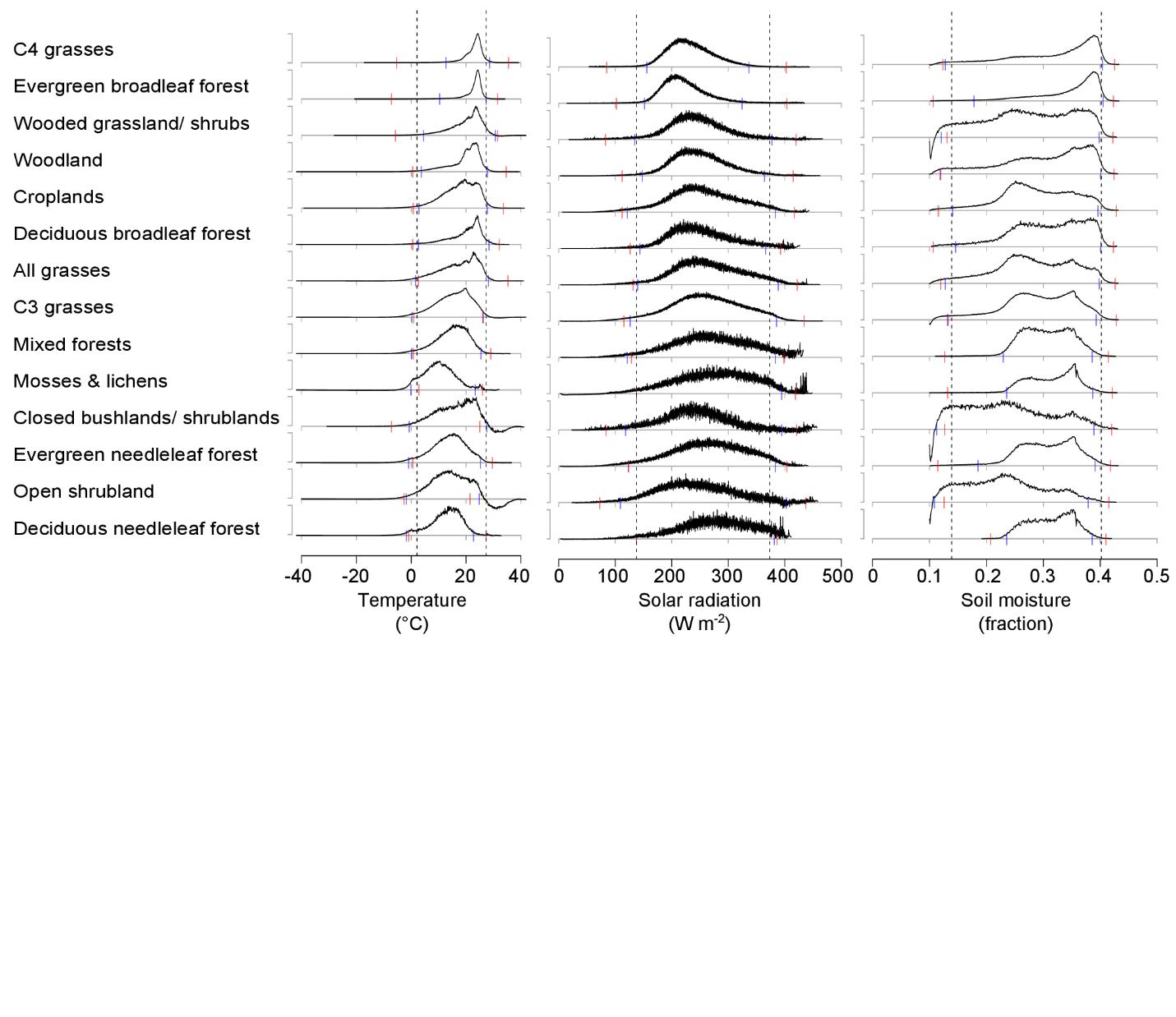

Supplement: S8 Fig — (DOCX) [file pbio.1002167.s017.docx]
