# Supplementary material for: Suitable Days for Plant Growth Disappear under Projected Climate Change: Potential Human and Biotic Vulnerability
Source: PLoS Biol. 2015 Jun 10;13(6):e1002167. doi: 10.1371/journal.pbio.1002167 (PMC4465630; doi:10.1371/journal.pbio.1002167)
Supplement: S1 Table — (DOCX) [file pbio.1002167.s018.docx]

**Table S1 | Earth System Models analyzed.** We only used models that included all three climatic variables at a daily resolution for at least one of three Representative Concentration Pathways and the Historical experiment. The variables analyzed include near-surface air temperature (CMIP5 variable name 'tas', in ^o^K), surface downwelling shortwave radiation (CMIP5 variable name 'rsds' in W m^-2^) and moisture in upper 10 cm portion of soil column (CMIP5 variable name 'mrsos' in kg m^-2^). Temperature was converted to ^o^C and soil moisture to volumetric water soil content using the following formula: VSM =SM/(R*rho), where VSM is volumetric soil water content (as a fraction), SM is soil moisture (in kg m^-2^), R is the depth of soil column (in m) and rho is water density (in kg m^-3^). These conversions were necessary to compare suitable plant growing days calculated from model projections and actual climate data (sources shown in Table S2). The results of this comparison indicate model accuracy and are shown in Figure S2.

| **CENTER** | **COUNTRY** | **MODEL** | **Historical** | **RCP 2.6** | **RCP 4.5** | **RCP 8.5** |
| --- | --- | --- | --- | --- | --- | --- |
| Beijing Climate Center, China Meteorological Administration | China | BCC-CSM1.1 | √ | √ | √ | √ |
| Canadian Centre for Climate Modelling and Analysis | Canada | CanESM2 | √ | √ | √ | √ |
| Commonwealth Scientific and Industrial Research Organization with Queensland Climate Change Centre of Excellence | Australia | CSIRO-Mk3.6.0 | √ | √ | √ | √ |
| College of Global Change and Earth System Science, Beijing Normal University | China | BNU-ESM | √ | √ | √ | √ |
| Institute for Numerical Mathematics | Russia | INM-CM4 | √ |  | √ | √ |
| Atmosphere and Ocean Research Institute (The University of Tokyo), National Institute for Environmental Studies, and Japan Agency for Marine-Earth Science and Technology | Japan | MIROC5 | √ | √ | √ | √ |
| Japan Agency for Marine-Earth Science and Technology | Japan | MIROC-ESM | √ | √ | √ | √ |
| Ocean Research Institute and National Institute for Environmental Studies | Japan | MIROC-ESM-CHEM | √ | √ | √ | √ |
| Met Office Hadley Centre | UK | HadGEM2-CC | √ |  | √ | √ |
|  |  | HadGEM2-ES | √ | √ | √ | √ |
| Meteorological Research Institute | Japan | MRI-CGCM3 | √ | √ | √ | √ |
| Norwegian Climate Centre | Norway | NorESM1-M | √ | √ | √ | √ |
| NOAA Geophysical Fluid Dynamics Laboratory | United States | GFDL-ESM2G | √ | √ | √ | √ |
|  |  | GFDL-ESM2M | √ | √ | √ | √ |
