# Supplementary material for: Suitable Days for Plant Growth Disappear under Projected Climate Change: Potential Human and Biotic Vulnerability
Source: PLoS Biol. 2015 Jun 10;13(6):e1002167. doi: 10.1371/journal.pbio.1002167 (PMC4465630; doi:10.1371/journal.pbio.1002167)
Supplement: S2 Table — (DOCX) [file pbio.1002167.s019.docx]

**Table S2 | Data sources**

| **Data and sources** | **URL** |
| --- | --- |
| **CMIP5 climate projections**  World Climate Research Programme*  **Observational climate data**  NCEP Reanalysis Daily Averages Surface Flux  Air temperature (2-m daily Mean)  Volumetric Soil Moisture 0-10 cm (Daily mean)  Downward Solar Radiation Flux (Daily mean) | http://pcmdi9.llnl.gov/esgf-web-fe/  http://www.esrl.noaa.gov/psd/cgi-bin/db_search/DBSearch.pl?Dataset=NCEP+Reanalysis+Daily+Averages+Surface+Flux&group=0&submit=Search |
| **Observed Net Primary Production data (modis)** | http://neo.sci.gsfc.nasa.gov/view.php?datasetId=MOD17A2_E_PSN |
| **Land use data**  Land cover types  C3 Grasses  C4 Grasses | http://webmap.ornl.gov/wcsdown/wcsdown.jsp?dg_id=10006_1  http://webmap.ornl.gov/wcsdown/wcsdown.jsp?dg_id=20042_6  http://webmap.ornl.gov/wcsdown/wcsdown.jsp?dg_id=20042_8 |
| **Map Outlines** | http://www.ngdc.noaa.gov/mgg/shorelines/shorelines.html  https://www.evl.uic.edu/pape/data/WDB/ |
| **VULNERABILITY ANALYSIS** |  |
| **Adaptability**  *per capita Gross Domestic Product*  Central Intelligence Agency of the United States | https://www.cia.gov/library/publications/download/download-2012/index.html |
| **Dependency** |  |
| *Food (Human consumption of NPP, % of country NPP)*  Human appropriation of Net Primary Production | http://sedac.ciesin.org/data/set/hanpp-by-country-and-product |
| *Revenue (Agriculture as % of GDP)*  Central Intelligence Agency of the United States | https://www.cia.gov/library/publications/download/download-2012/index.html |
| *Jobs (Agriculture jobs as % of working force)*  Central Intelligence Agency of the United States | https://www.cia.gov/library/publications/download/download-2012/index.html |
| *Complementary data sources***  Food and Agriculture Organization of the United Nations | http://www.fao.org/docrep/018/i3107e/i3107e01.pdf |
| World Bank Database | http://www.data.worldbank.org |
| Institute of issuing overseas departments (IDEOM -France) | http://www.iedom.fr/iedom |
| National Institute of Statistics and Economic Studies (France) | http://www.insee.fr/fr/publications-et-services/default.asp |
| United Nations Database | http://data.un.org |
| States Of Guernsey Government web portal | http://www.gov.gg |
| Australian Bureau of Statistics | http://www.abs.gov.au/websitedbs/censushome.nsf/home/data |
| Internal Revenue Service of the United States | http://www.irs.gov/Individuals/International-Taxpayers |
| Portuguese Census Data 2011 | http://www.ine.pt/scripts/flex_v10/Main.html |
| States of Jersey Government Web Portal | http://www.gov.je/Government/Pages/StatesReports.aspx |
| AICEP - Business Development Agency Portugal | http://www.portugalglobal.pt |
| University of the South Pacific | http://www.usp.ac.fj/index.php?id=2538 |
| Statistics Agency of the Government of Niue | http://www.spc.int/prism/niue/index.php/niue-documents |
| Central Agency for Statistics (Netherlands) | http://www.cbs.nl/en-GB/menu/publicaties/default.html |
| Central Bureau of Statistics of Curacao | http://www.cbs.cw |
| Index Mundi | http://www.indexmundi.com |

*We acknowledge the World Climate Research Programme’s Working Group on Coupled Modeling, which is responsible for CMIP5, and we thank the climate modeling groups (listed in Table S1 of this paper) for producing and making available their model output. The CMIP is the U.S. Department of Energy’s Program for Climate Model Diagnosis and Intercomparison, which also provides coordinating support and leads development of software infrastructure in partnership with the Global Organization for Earth System Science Portals.

**Data on country level population, “Revenue” and “Jobs” were compiled primarily from The Central Intelligence Agency World Factbook and, when incomplete, “Complementary data sources” were used to fill gaps.
