# Supplementary material for: Suitable Days for Plant Growth Disappear under Projected Climate Change: Potential Human and Biotic Vulnerability
Source: PLoS Biol. 2015 Jun 10;13(6):e1002167. doi: 10.1371/journal.pbio.1002167 (PMC4465630; doi:10.1371/journal.pbio.1002167)
Supplement: S3 Table — (DOCX) [file pbio.1002167.s020.docx]

**Table S3 | Human vulnerability to changes in suitable days for plant growth.** Values indicate numbers of people (as of 2012 in billions) in countries categorized by exposure, dependency and adaptability. “Exposure” was categorized as countries having high loss (HL), medium loss (ML), no change (NC), medium gain (MG), and high gain (HG) in suitable days in the last decade of 2100 compared to the number of contemporary suitable days under three RCPs. Dependency was categorized as countries with low (L), medium (M), or high (H) dependency on plant-related goods and services. Adaptability was categorized as countries with low (L), medium (M), and high (H) income. See Methods for category definitions.

| **Exposure** | **Dependency** | **Adaptability** | **RCP 2.6** | **RCP 4.5** | **RCP 8.5** |
| --- | --- | --- | --- | --- | --- |
| **HL** | **H** | L |  |  | 0.022 |
|  |  | M |  |  | 0.424 |
|  |  | H |  |  | 0.036 |
|  | **M** | L |  |  |  |
|  |  | M |  |  | 0.046 |
|  |  | H |  |  |  |
|  | **L** | L |  |  |  |
|  |  | M |  |  |  |
|  |  | H |  |  | 0.004 |
| **ML** | **H** | L |  | 0.015 | 2.109 |
|  |  | M | 0.085 | 0.191 | 0.229 |
|  |  | H |  | 0.035 | 0.07 |
|  | **M** | L |  |  | 0.001 |
|  |  | M |  |  | 0.086 |
|  |  | H |  | 0.002 | 0.003 |
|  | **L** | L |  |  |  |
|  |  | M | 0.002 | 0.004 | 0.122 |
|  |  | H | 0.006 | <0.001 | 0.26 |
| **NG** | **H** | L | 2.732 | 2.753 | 0.625 |
|  |  | M | 1.945 | 1.839 | 1.377 |
|  |  | H | 0.934 | 0.898 | 0.822 |
|  | **M** | L | 0.001 | 0.001 |  |
|  |  | M | 0.206 | 0.206 | 0.071 |
|  |  | H | 0.53 | 0.528 | 0.527 |
|  | **L** | L |  |  |  |
|  |  | M | 0.12 | 0.118 | 0.001 |
|  |  | H | 0.487 | 0.494 | 0.048 |
| **MG** | **H** | L | 0.037 | 0.001 | 0.013 |
|  |  | M |  |  |  |
|  |  | H |  | 0.003 | 0.008 |
|  | **M** | L |  |  |  |
|  |  | M |  |  | 0.003 |
|  |  | H |  |  |  |
|  | **L** | L |  |  |  |
|  |  | M |  |  |  |
|  |  | H | 0.001 | <0.001 | 0.182 |
| **HG** | **H** | L |  |  |  |
|  |  | M |  |  |  |
|  |  | H | 0.002 |  |  |
|  | **M** | L |  |  |  |
|  |  | M |  |  |  |
|  |  | H |  |  |  |
|  | **L** | L |  |  |  |
|  |  | M |  |  |  |
|  |  | H |  |  | <0.001 |
